# Supplementary material for: Social validity of acceptance-based workplace mental health training for use in a low resource setting. A qualitative study with Ugandan mental health providers
Source: PLOS Ment Health. 2024 Sep 20;1(4):e0000127. doi: 10.1371/journal.pmen.0000127 (PMC12798340; doi:10.1371/journal.pmen.0000127)
Supplement: S1 Text — (PDF) [file pmen.0000127.s001.pdf]

ACT for the Workplace Intervention.

Interview guide for Social validity

Version 1

Interview guide for assessing social validity

1. How do feel about the program? Is it relevant? If YES/NO, why? Can it work in Uganda? What elements did you identify that you think may not be applicable in Uganda? Why do you think they are not applicable? What is the goal of the program? Does it make sense to you?
2. How easy was it for you to keep following? Are there moments where you felt confused? Or wondered about what is going on? If Yes, what caused the confusion most? Do you think the way the program is delivered is appropriate for use in Uganda? Why? Were the procedures easy to follow? Why? will other people also find the program easy to understand? Were you comfortable with the exercises, examples of games used in the program?
3. Do you think the program is sufficient to provide you skills to deal with daily life challenges? How important will it be to add this program to employee assistance programs at work? Would you recommend someone into this program? Why?
4. In your opinion, what can be done to make the program better?
