# Supplementary material for: Social validity of acceptance-based workplace mental health training for use in a low resource setting. A qualitative study with Ugandan mental health providers
Source: PLOS Ment Health. 2024 Sep 20;1(4):e0000127. doi: 10.1371/journal.pmen.0000127 (PMC12798340; doi:10.1371/journal.pmen.0000127)
Supplement: S1 Data — (DOCX) [file pmen.0000127.s002.docx]

**Code book for social validity of ACT for the workplace**

| **Social validity ‘*priori theme’*** | **Theme description** | **Emerging code** | **Relatable quotes** |
| --- | --- | --- | --- |
| Socially valid Goals | Are the behavioral goals being achieved by the intervention what the society really needs? | ‘Nature of goals achieved by ACT’ | ‘Program is mainly focused on the individual achieving personal goals, yet Uganda is a collective society’ AW002  ‘Community activities or cooperative tasks, where individuals work together to achieve a common goal while acknowledging challenges are more appropriate’ AW001  ‘Focusing solely on individual values and goals may not be as well-received in a collectivist culture’ AW005 |
|  |  | Importance of ACT goal | ‘The program is related to wellbeing of people which is an important aspect in Ugandan workplaces’AW004  ‘The aim of the training is to equip you with skills to manage yourself even when the situation is disturbing. Many Ugandans will like this because a lot is going on’ AW011  ‘Mental health is not well understood here, many people still relate it to witchcraft and personal problems. A program of this nature should include awareness and fighting mental health stigma as part of the goals, this makes it more relevant AW0010  ‘ACT gives practical tools to navigate your life. It will not solve poverty problems but helps one to look at their situation differently, this is important’ AW005 |
|  |  | Acceptance of practice | Some individuals and communities in Uganda have deep-rooted beliefs that prioritize supernatural explanations for mental health and are skeptical of Western therapeutic approaches’ AW006  ‘Psychotherapy in Uganda has been around long enough but not fully accepted. It’s often mocked by people who think spirituality or traditional approaches offer quick solutions’ AW010  ‘ACT encourages allowing uncomfortable feelings to be experienced while leading a fulfilling life, this connects well with religious teachings and thus Ugandans will believe it’ AW007 |
|  |  | Relevance of MH | ‘Workplaces here are full of men. Men in Africa downplay mental health issues and look at approaches focused on wellness as wastage of time. Mental wellness is always getting less attention’  ‘Since COVID time, that has been increase awareness of mental health especial in workplaces, Ugandans are starting to look as mental wellness as important’ AW014 |
| Socially appropriate procedures | Are the methods of delivery, approaches and mechanism of change used by ACT feasible and acceptable to the targeted society? | ‘Clarity of language’ | ‘The language of the program is a bit more advanced with technical jargons, it may not be understood. AW014  ‘Given that English serves as the official language in Uganda and used in most workplaces, the program will be good for high levels workers who speak English not casual workers who speak local languages’ AW09 |
|  |  | ‘Incongruent metaphors’ | ‘Some metaphors will be hard to figure out by people here, it would be better if we use traditional Ugandan game or activity that involves cooperation and competition, highlighting the balance between accepting emotions and taking purposeful actions’ AW009  ‘Metaphor with a natural element such as the vastness of the African savannah or the horizon over Lake Victoria would be more appreciated not the ones used here like ‘eating raisins’ AW003  ‘This approach does not incorporate traditional wisdom or proverbs that highlight the value of accepting challenges to grow stronger. It would make sense if it Integrated storytelling featuring local heroes who faced adversity with courage, illustrating how’ AW005 |
|  |  | Time required | ‘Two hours per session is a lot of time which organizations in Uganda cannot give’ AW006  ‘Most organizations in Uganda are understaffed and people work long hours, no one can give you all that time to attend sessions’ AW006 |
|  |  | Conflicting with spirituality | ‘Mindfulness is more of Buddhism which contradicts the strong religion links Ugandans have with Christianity and Islam’ AW003  ‘ACT does not appreciate that certain things that happen to people are decided by God and its him to offer solutions. It has assumed that people can get along with everything’ AW14  ‘It is important to ensure that the ACT program is respectful of religious beliefs, possibly integrating elements of spirituality where it aligns with the overall objective of the training’ AW08 |
|  |  | Structure of the program | The step-by-step breakdown of each session, coupled with detailed explanations and experiential exercises makes understanding easier’ AW001  ~~‘The training starts with mindfulness, which is not a common practise in Uganda. This easily creates bias about the entire program’ AW006~~  ‘  ‘Flexibility of the program will make it easy to implement. No need to cram things, ACT feels like naturally flowing, people will like it. Doesn’t burden like other work training programs’ AW011  ‘The program is structured and requires people to attend several sessions. In Ugandan workplaces, mental health talks are one day events which require less commitment of resources. Both employers and employees may not commit to such a structured training’ AW005  ‘Program requires a lot of reading, Ugandans just don’t like reading much’ AW007 |
|  |  | Process of change | ACT feels like a life experience. People just scan through their lives and make personal choices. You find your own solutions basing on what works for you. This self-discovery is important. Ugandans are so used to being told what to do and this has not helped much’ AW005 |
|  |  | Collective activities | ‘Most things in this program rotates about telling individuals to do this and that, in Uganda it’s about groups, teams, colleagues and collective activities’ AW007  ‘Program should have focused on telling participants to identify shared values and collaboratively set group goals that contribute to the well-being of the entire team’, this can then be followed with the individual’ AW008 |
|  |  | Group processes | ‘The proposed group composition in the manual may not fully reflect the diversity and social dynamics of Ugandan workplaces’ AW007  ‘Factors such as hierarchy, gender roles, and power distance influence interactions and discussions within groups and should be considered’ AW007 |
|  |  | Power dynamics | ‘Ugandan workplaces have distinct hierarchical structures that can impact how employees interact with each other and with facilitators. This should be taken into account during training planning’ AW001  ‘Lower cadre employees never express themselves in presence of bosses’ AW006 |
|  |  | Content and concepts | ‘Program over emphasizes psychology, in Uganda psychology is about mind reading’ AW007  ‘Mindfulness exercises that require closing eyes will not be taken serious, people will be laughing’ AW009  ‘The program is about the workplace, but its connection with the workplace is minimal, people will keep asking’ AW0011  The program uses advanced psychology terms and assumes an audience with some knowledge about psychology or mental health. Ugandans are no knowledgeable in such areas’  ‘The training is about acceptance, this means surrendering which is a sign of weakness, Ugandans are raised to be brave to deal with challenges’ AW0011 |
|  |  | Facilitators | ‘Program suggest facilitators with specific professional backgrounds that may be scarce in Uganda’ AW001  Qualities that are highly valued within the local context, such as humility, respect, and strong interpersonal skills should be the main criteria of consideration for facilitators’ AW001 |
|  |  | Believability of treatment process | ‘Ugandans believe in practical traditional healing practices, yet ACT relies on just talking about the problem. It will be hard for people to believe that talking will make things any better’  ‘Traditional beliefs and healing methods such as witchcraft, spirituality and herbal remedies are highly valued when talking about wellness and mental health. ACT is western psychotherapy which is blind to all this’ |
| Intervention Outcomes | The relevance of the behavior being changed. | Health enhancement | ‘Of recent we see many people in Uganda breaking down and dying due to stress. A program that promotes mental wellness is timely; AW0011  ‘Living in a poor country is a big challenge, everything is a struggle so since this program helps people learn how to live with their problems, it will result in better health and fewer worries’ AW004  ‘It is so practical. Just going through it, I am already feeling relieved as an individual. Others will also benefits from it’ AW007 |
|  |  | Improved productivity | ‘We all know that mental health reduces productivity of individuals, this program is training people to manage their own mental health. I am sure they will turn out to be more productive for themselves and organizations’. AW004 |
