# Supplementary material for: Social validity of acceptance-based workplace mental health training for use in a low resource setting. A qualitative study with Ugandan mental health providers
Source: PLOS Ment Health. 2024 Sep 20;1(4):e0000127. doi: 10.1371/journal.pmen.0000127 (PMC12798340; doi:10.1371/journal.pmen.0000127)
Supplement: Attachment — Submitted filename: Response to reviewer.docx [file pmen.0000127.s003.docx]

11^th^ June 2024

**Re-submission of " Social validity of acceptance-based workplace mental health training for use in a low resource setting. *A qualitative study with Ugandan mental health providers.* PMEN-D-24-00104**

Dear Editor-in-Chief, PLOS MENTAL HEALTH

Thank you for the opportunity to revise and resubmit our manuscript. I also thank the reviewer for the constructive feedback which has helped to improve the quality of the manuscript. We have given careful attention to the comments and I believe the manuscript more closely aligns to the PLOS MENTAL HEALTH publication criteria. Responses to reviewer’ comments are presented in the table below:

| **Review comment** | **Response** |
| --- | --- |
| The data was analyzed by the writers using a deductive thematic analysis approach. Nevertheless, no particular literature or theory is offered in the method section to clarify the meaning of a deductive thematic approach or the reasons it might be preferred to other approaches. This lack of backing calls into question the rationale and preferences for this kind of approach. | We appreciate this observation. Additional literature justifying the use of a deductive analysis approach has been added. See lines 288-300 and lines 205-211 |
| The approach description is included in the theme analysis together with the literature review, but this is not enough support. Before producing an extensive literature review with citations from the most recent to the earlier studies, authors must first distinguish the introduction from the literature review. | We agree with the reviewer that separating the introduction and literature review would make the manuscript stronger. We have addressed this by separating the two sections. See line 62-108 |
| Authors must first distinguish the introduction from the literature review in order to improve the support offered by these two techniques. The introduction gives a broad background for the research as well as a succinct summary of the subject. It does not, however, particularly address the goals or research questions that will direct the literature review. Writing the introduction and literature review separately allows authors to make sure each piece does what it is supposed to do. | As acknowledged above, the two sections have been separated. |
| Writers can concentrate on producing an in-depth literature evaluation after separating the introduction from the review. A comprehensive literature review entails a detailed analysis of the body of existing research on the subject of interest. To give a thorough grasp of the study field, it entails finding pertinent studies, evaluating their major conclusions, and synthesizing this data. | Thank you for the guidance, this has been addressed in depth as earlier noted. See lines 62-191 |
| Authors should cite a variety of sources, both historical and contemporary, to guarantee a comprehensive literature assessment. Authors can provide the groundwork for future study and expand on what is already known in the field by utilizing citations from earlier studies. On the other hand, authors can remain current with the most recent advancements and theories in the field by incorporating citations from recent research. Writing from both historical and modern viewpoints allows authors to present a complete picture of the research issue. | A comprehensive literature assessment has been done and several current sources cited. See lines 108-157 |
| Authors should also assess the literature they include in the review critically. This entails evaluating the research's quality, taking into account its methodology, and determining the accuracy and dependability of the data it offers. Authors can make sure that the review offers trustworthy support for the research being done by thoroughly analyzing the literature. | Thank you. The literature reviewed and cited in the study has been comprehensively assessed for quality as showed in the introduction and literature review sections. Lines 62-191. |

Sincerely,

Khamisi Musanje

Corresponding author
