# Supplementary material for: Social validity of acceptance-based workplace mental health training for use in a low resource setting. A qualitative study with Ugandan mental health providers
Source: PLOS Ment Health. 2024 Sep 20;1(4):e0000127. doi: 10.1371/journal.pmen.0000127 (PMC12798340; doi:10.1371/journal.pmen.0000127)
Supplement: Attachment — Submitted filename: Response to reviewer.docx [file pmen.0000127.s004.docx]

15^th^ August 2024

**Re-submission of " Social validity of acceptance-based workplace mental health training for use in a low resource setting. *A qualitative study with Ugandan mental health providers.* PMEN-D-24-00104**

Dear Editor-in-Chief, PLOS MENTAL HEALTH

Thank you for the opportunity to revise and resubmit our manuscript for the second time. I also thank the reviewer for the constructive feedback which has helped to improve the quality of the manuscript. We have given careful attention to the comments and I believe the manuscript more closely aligns to the PLOS MENTAL HEALTH publication criteria. Responses to the comments are presented in the table below:

| **Review comment** | **Response** |
| --- | --- |
| Please address the section on line 45 re deductive analysis. It is not clear how the analysis was deductive, why not inductive given the qualitative participatory nature of the interviews with the workers? | Thank you for this observation. The analysis was deductive since from the design of the interview guide, to the collection of data and analysis, we were working through lenses of the Social validity framework. During analysis, “*codes were organized according to a priori thematic categories that aligned with the Social Validity Framework*” This has been clarified in the manuscript on line 45-46. |
| Please clarify line 205 where you discuss "lense of a framework" do you mean social validity framework as in line 516? | Thank you for the comment. Yes, in line 205 we are referring to lenses of the social validity framework. This has been clarified in line 206 |

Sincerely,

Khamisi Musanje

Corresponding author
